# Prophylactic Laparoscopic Total Gastrectomy with Jejunal Pouch Reconstruction in Patients Carrying a CDH1 Germline Mutation

**DOI:** 10.1007/s11605-015-2963-4

**Published:** 2015-10-06

**Authors:** L. Haverkamp, P.C. van der Sluis, M.G.E.M. Ausems, S. van der Horst, P.D. Siersema, J.P. Ruurda, G.J.A. Offerhaus, R. van Hillegersberg

**Affiliations:** Department of Surgery, University Medical Center Utrecht, Heidelberglaan 100, 3584 CX Utrecht, The Netherlands; Department of Medical Genetics, University Medical Center Utrecht, Heidelberglaan 100, 3584 CX Utrecht, The Netherlands; Department of Gastroenterology and Hepatology, University Medical Center Utrecht, Heidelberglaan 100, 3584 CX Utrecht, The Netherlands; Department of Pathology, University Medical Center Utrecht, Heidelberglaan 100, 3584 CX Utrecht, The Netherlands

**Keywords:** Gastrectomy, Laparoscopy, Prophylactic, E-cadherin, Cancer

## Abstract

**Background:**

For patients with an identified germline E-cadherin-1 (CDH1) mutation, prophylactic gastrectomy is the treatment of choice to eliminate the high risk of developing diffuse gastric cancer. Laparoscopic total gastrectomy with jejunal pouch reconstruction is a novel approach that may be especially suitable in these patients.

**Methods:**

Patients with a germline CDH1 mutation who underwent prophylactic laparoscopic total gastrectomy with jejunal pouch were included in our prospective database.

**Results:**

A total of 11 patients with a median age of 40 (22–61) years were included. The average operative time was 4:26 ± 0:49 h and the average blood loss was 219 ± 155 ml. Median length of hospital stay was 10 (7–27) days. In two patients, an esophagojejunal anastomotic leakage occurred (grade 4). The leakages were seen in patient numbers 2 and 3, which may be a result of a learning curve. The latter eight patients did not develop anastomotic leakage. Pulmonary complications occurred in one patient with atelectasis and in one patient with pneumonia (grade 2). The 60-day mortality rate was 0 %. Multiple foci of intramucosal diffuse gastric signet ring cell carcinoma were found in the resection specimen of 9/11 (82 %) patients. All 11/11 (100 %) resections were microscopically radical.

**Conclusions:**

Prophylactic laparoscopic total gastrectomy with jejunal pouch reconstruction in patients with a CDH1 germline mutation is feasible and safe. In 82 % of patients, foci of intramucosal diffuse gastric signet ring cell carcinoma in the resection specimen were found.

## Introduction

The estimated number of newly diagnosed gastric cancer patients is 989,600 worldwide, accounting for 738,000 cancer-related deaths.^1^ This number counts for 10 % of all cancer-related deaths, ranking gastric cancer the second highest cause of cancer death.^1^

Hereditary diffuse gastric cancer represents 1–3 % of all gastric cancers.^2^ In approximately 30 % of families with hereditary diffuse gastric cancer, germline mutations are found in the E-cadherin-1 (CDH1) tumor suppressor gene.^3^–^6^ The CDH1 gene codes for the E-cadherin protein, and loss of function of the CDH1 gene leads to diffuse gastric cancer. Patients who carry CDH1 germline mutations have a lifetime risk of >70 % of developing diffuse gastric cancer with a 5-year survival of less than 20 %.^7^,^8^ Female carriers also have an increased risk to develop lobular breast cancer.^9^–^11^ Surgery eliminates the high risk of developing diffuse gastric cancer in patients with germline CDH1 mutations and therefore is the treatment of choice. Most reports on prophylactic total gastrectomy in literature describe a conventional open surgical approach.^12^

In gastric cancer, laparoscopic total gastrectomy showed diminished blood loss, fewer postoperative complications, and shorter postoperative hospital stay.^13^ This technique may therefore be especially suitable for prophylactic surgery. In this article, we describe our initial experience with prophylactic laparoscopic total gastrectomy with jejunal pouch in a case series of patients carrying a CDH1 germline mutation.

## Materials and Methods

### Patients

Patients (*n* = 11) with identified germline CDH1 mutations who were referred to the University Medical Centre Utrecht for prophylactic total gastrectomy between April 2006 and May 2015 were included in this study. All patients were presented and discussed in a multidisciplinary team meeting comprising oncologic surgeons, medical oncologists, gastroenterologist, clinical geneticist, radiologists, and nutritionist prior to treatment. Patient information was recorded into a prospectively maintained database and included demographics, preoperative clinical work-up, surgical results and postoperative complications, pathological results, length of hospital stay, and follow-up. No routine upper gastrointestinal contrast studies were performed to detect anastomotic leakage, instead radiological diagnosis was performed when clinical signs of leakage were present. Complications were scored with the modified Clavien-Dindo classification.^14^

### Surgery

**Fig. 1:**
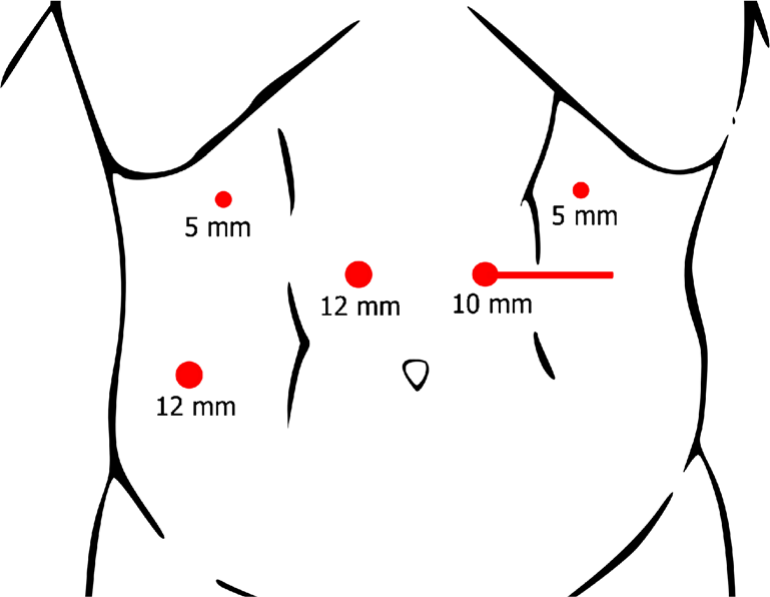
Trocar arrangement during prophylactic laparoscopic total gastrectomy. The camera was inserted through the 10-mm para-umbilical trocar port and two 5-mm trocars were used as laparoscopic working ports. The liver retractor was inserted through the 12-mm right para-rectal trocar port. The harmonic scalpel was inserted through the 12-mm para-umbilical port. The *horizontal line* represents the incision that is used to take out the stomach

**Fig. 2:**
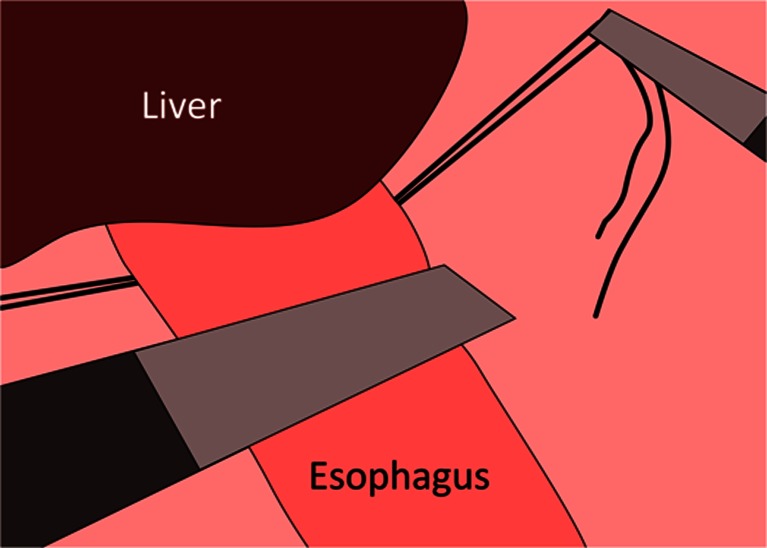
Placement of a supporting suture on both sides of the esophagus followed by transection of the esophagus by means of an Endo-GIA stapler

All patients underwent prophylactic laparoscopic total gastrectomy by surgeons experienced in laparoscopic techniques (RvH and JPR). All patients received an epidural catheter to provide adequate postoperative analgesia. Prophylactic antibiotics were administered pre-operatively. After an open introduction of a first trocar in the left hypogastrium, pneumoperitoneum was created and the abdominal cavity was inspected, and two 5-mm trocars were introduced subcostally on both sides and two 12-mm trocars in the right hypogastrium and the right flank under direct sight (Fig. 1). The lesser sac was opened and transected closely to the liver. The left crus of the diaphragm was exposed. Hereafter, the greater gastric curvature was dissected using a harmonic scalpel (Ethicon Endosurgery, Southington, CT, USA) followed by dissection and ligation of the left gastric artery and vein. The right gastroepiploic artery and vein were dissected and ligated at the level of the duodenum. The duodenum was divided postpyloric with an Endo-GIA stapler (Covidien, Norwalk, CT, USA). The esophagus was mobilized, and a supporting suture was placed on both sides followed by transection of the distal esophagus with an Endo-GIA stapler (Fig. 2). An Orvil stapler (Covidien, Norwalk, CT, USA) anchor was introduced through the oral cavity into the esophagus. In the stapled distal esophagus, an opening was created for the Orvil anchor, which was fixed by a laparoscopically placed purse-string suture around the anchor.

**Fig. 3:**
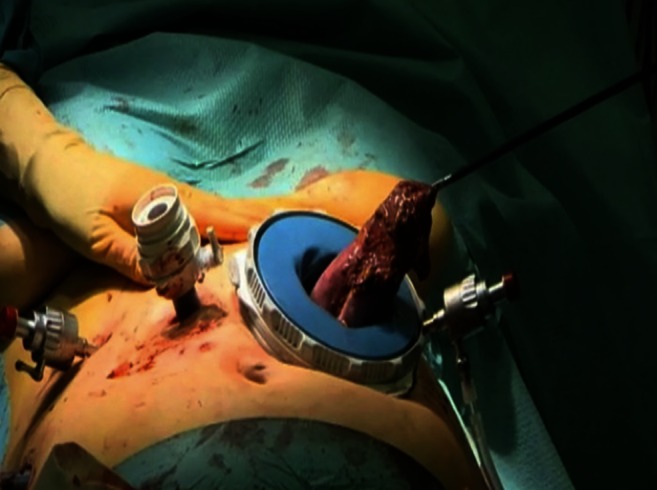
Removal of the gastric resection specimen through the Endopath Dextrus^TM^ access port

**Fig. 4:**
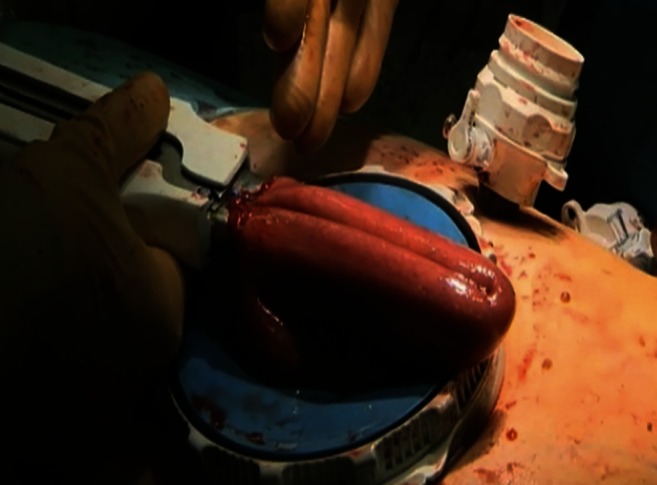
Creation of a “J”-shaped jejunal pouch, using a 100-mm linear stapler

**Fig. 5:**
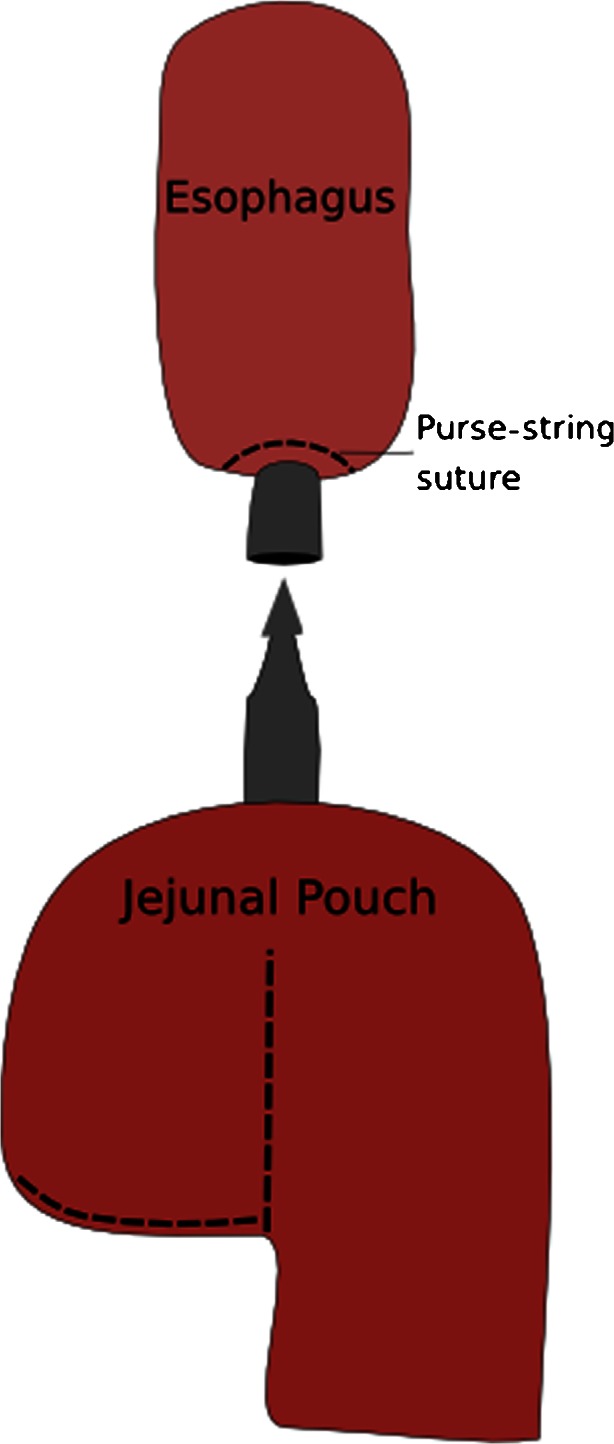
Creation of an esophagojejunal anastomosis with jejunal pouch with the use of the Orvil circular stapler

A horizontal incision in the left upper abdomen, with sparing of the rectus muscle, was created through the left 12-mm port, and an Endopath Dextrus^TM^ (Ethicon Endosurgery, Southington, CT, USA) access port was inserted. The resection specimen was removed through this port and was sent in for frozen section evaluation (Fig. 3). The first bowel loop was divided with an Endo-GIA stapler, and the Roux-en-Y reconstruction was completed with an isoperistaltic side-to-side jejunal-jejunal anastomosis manually created with a PDS 3.0 continuous suture. The distal jejunum was used to create a “J”-shaped pouch (Fig. 4), using a 100-mm linear stapler (Covidien, Norwalk, CT, USA). An antecolic 10-cm esophageal (pouch)-jejunal (EJ) anastomosis was created with the Orvil circular stapler (Fig. 5). The proximal and distal esophageal-jejunal donuts were pathologically examined. The blind jejunal loop was stapled. Distal to this anastomosis, a feeding jejunostomy was inserted.

### Pathological Analysis

The resection specimen was evaluated using a standard protocol, providing information on resection margins and presence of tumor cells. The specimen was completely accessioned; after resection of the proximal and distal margin, the remaining mucosa was cleaved from the underlying deep muscle and fixed overnight according to the so-called Swiss roll technique.^15^ This method consists of isolating a thin strip of gastric mucosa (approximately 4 mm thick) from the underlying layers and rolling it up in a spiral starting from the distal (duodenal) margin. This enables complete microscopic investigation of whole gastric mucosa and is particularly suitable when no macroscopic lesions can be recognized.^15^

## Results

A total of 11 patients (eight females, three males) who carried the CDH1 gene mutation were included and underwent prophylactic laparoscopic total gastrectomy. The median age at the time of surgery of was 40 (22–61) years (Table 1). All patients had an ASA score of 1, 2, or 3. The average operative time was 4:26 ± 0:49 h (Table 2). The average blood loss was 219 ± 155 ml. No blood transfusions were required. One procedure was converted to an open procedure due to an incomplete anastomosis identified during the procedure, which necessitated additional stitches. The median length of postoperative hospital stay was 10 (7–23) days.

**Table 1:** Baseline characteristics

	Laparoscopic prophylactic gastrectomy
	*N* = 11
Age^a^	40 (22–61)
Gender (male/female)	3:8
BMI^a^	27.8 (19.7–35.2)
Comorbidities	
Diabetes mellitus	0
COPD	0
Cardiovascular	1
ASA score	
1	6
2	4
3	1
Smoking	6
Alcohol	5

^a^Data presented as median (min–max)

**Table 2:** Intraoperative and postoperative outcomes

	Laparoscopic prophylactic gastrectomy
	*N* = 11
Duration of surgery (h)^a^	4:26 ± 0:49
Blood loss (ml)^a^	219 ± 155
Conversion	1
Complicated course	5
Postoperative hospital stay (day)^b^	10 (7–23)
Intensive care stay (day)^b^	0 (0–2)
Resected lymph nodes (*n*)^b^	10 (1–25)
R0 resection	11
Mortality	
30-day mortality	0 %
60-day mortality	0 %

^a^Average ± standard deviation

^b^Median (min–max)

In two patients, anastomotic leakage occurred which were treated either by intraoperative anastomotic repair or endoscopic placement of a stent (grades 3–4). Both leakages were detected after surgery. No intraoperative leak test was routinely performed. The first patient was stented due to fistula formation at the level of the anastomosis, and the second patient underwent a re-operation during which an anastomotic dehiscence was seen. This defect was oversewn after which the patient recovered uneventfully. The leakages were seen in patient numbers 2 and 3, which may be a result of a learning curve. The latter eight patients did not develop anastomotic leakage. Minor complications were observed in two patients. One patient suffered from atelectasis and a wound infection, which were treated conservatively (grade 1). One of these patients had a pneumonia, which was treated with intravenous antibiotics (grade 2). The remaining four patients had no early postoperative complications (<30 days postoperatively).

**Fig. 6:**
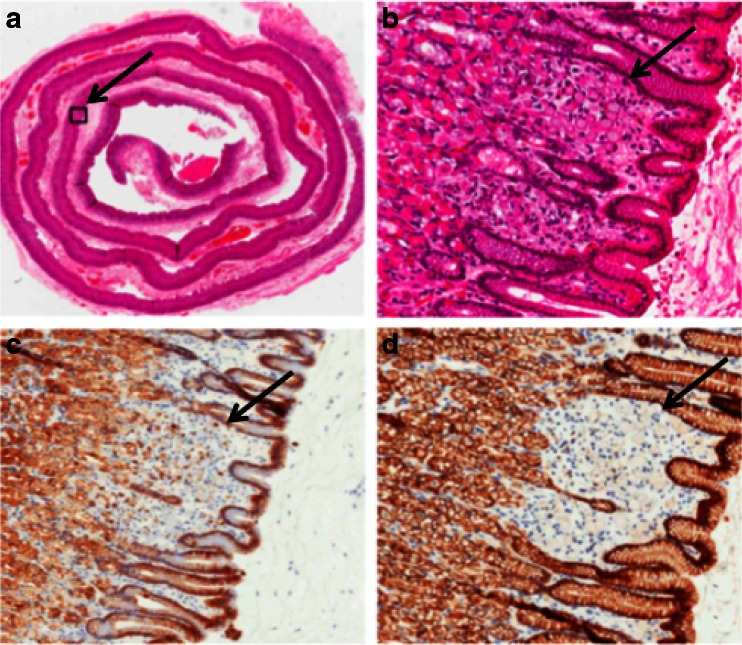
Swiss roll with signet cell in HE staining (**a, b**), CAM5.2 staining (**c**), and E-cadherin immunohistochemistry (**d**)

After microscopy of the complete resection specimen, multiple foci of intramucosal diffuse gastric signet ring cell carcinoma or focal intestinal metaplasia were found in the resection specimen of 9/11 (82 %) patients (Fig. 6). The largest focus was 8 mm in diameter. A median of 10 (1–25) lymph nodes were dissected during laparoscopy, none of which were tumor positive.

## Discussion

In this article, we report our initial experience with prophylactic laparoscopic total gastrectomy with jejunal pouch in patients with a CDH1 germline mutation. In 9/11 (82 %) patients, multiple foci of intramucosal diffuse gastric signet ring cell carcinoma were found in the resection specimens. The laparoscopic technique was shown to be feasible and safe with an average blood loss of 219 ± 155 ml and median length of hospital stay of 10 days. Postoperative complications occurred in 6/11 (55 %) patients, and the 60-day hospital mortality rate was 0 %.

Our prophylactic gastrectomy resection specimens were microscopically analyzed using the “Swiss roll” technique. In this fashion, the complete gastric mucosa could be scrutinized by expert pathologists.^15^ In our cohort, multiple foci of intramucosal gastric cancer were found in the resection specimens in 82 % of patients. This is in concordance with other series, where the presence of one or more foci of signet ring cell carcinoma was reported in up to 92 % of all prophylactic gastrectomy pathologic resection specimens.^3^,^16^,^17^ The high percentage of intramucosal gastric cancer in both our series and the series from literature emphasizes the need for prophylactic gastrectomy for patients with germline CDH1 mutations.

In a meta-analysis, we demonstrated that laparoscopic total gastrectomy is associated with reduced intraoperative blood loss, lower risk at postoperative complications, and shorter hospital stay compared to open total gastrectomy in patients with gastric cancer.^13^ These benefits are especially relevant for patients with a CDH1 germline mutation. Our initial experience with the prophylactic laparoscopic total gastrectomy is promising. We expect that the outcomes of prophylactic laparoscopic total gastrectomy will approximate the results for laparoscopic total gastrectomy for gastric cancer in larger series of patients with a CDH1 germline mutation.

The median length of hospital stay in our cohort was 10 days. This is higher than reported in a case report of patients that had prophylactic laparoscopic total gastrectomy (median length of hospital stay was 5 days).^18^ This might be due to the fact that this was our initial experience, and these results may have been part of our learning curve. According to literature, the learning curve for total gastrectomy is 23 procedures.^19^ The current study included 11 patients, so an actual learning curve was not analyzed. Also, in our series, all patients were placed on a nil-by-mouth routine with enteral tube feeding by a needle-catheter jejunostomy the first 7 days postoperatively. Results may be further improved by implying a fast recovery protocol with early start on clear liquid diets and early mobilization.^20^

A D1 lymph node dissection was performed in this cohort of patients according to the international guidelines.^21^ We found that the laparoscopic procedure yielded a median of 10 lymph nodes. All dissected lymph nodes were negative for metastases. This is in accordance with literature describing the removal of 10–12 lymph nodes, none of which contained metastases.^12^ Until now, after a median follow-up time of 28 (9–110) months, no recurrent disease or distant metastases were observed in our cohort, suggesting that a limited lymph node dissection is the appropriate procedure for these patients.

Compared to open total gastrectomy for gastric cancer, laparoscopic total gastrectomy showed less blood loss, fewer postoperative complications, and shorter hospital admission time as well in this series as in literature.^13^ Most probably, this is the result of less surgical trauma. The severity of surgical trauma can be assessed by measuring the serum levels of the acute-phase response cytokine IL-6. A recent systematic review indicated that an open procedure is associated with a higher inflammatory response, as is measured by IL-6 levels compared to a laparoscopic procedure.^22^ Unlike laparoscopic distal gastrectomy, laparoscopic total gastrectomy is still not widely accepted as first choice of treatment. This is probably a reflection of laparoscopic total gastrectomy being a technically more demanding procedure than laparoscopic distal gastrectomy with a long learning curve.^23^

## Conclusion

In conclusion, we showed that prophylactic laparoscopic total gastrectomy with jejunal pouch for patients with germline CDH1 mutation is feasible and safe. Laparoscopic total gastrectomy is associated with less intraoperative blood loss, fewer postoperative complications, and shorter hospital admission time in comparison to open total gastrectomy. These benefits may be especially relevant to patients with germline CDH1 mutation. We therefore recommend experienced surgeons to consider prophylactic laparoscopic total gastrectomy as first choice for these patients.
